# Supplementary material for: The Need to Set up a Biobank Dedicated to Lymphoid Malignancies: Experience of a Single Center (Laboratory of Clinical and Experimental Pathology, University Côte d’Azur, Nice, France)
Source: J Pers Med. 2023 Jun 29;13(7):1076. doi: 10.3390/jpm13071076 (PMC10381579; doi:10.3390/jpm13071076)
Supplement: Supplementary file 1 [file jpm-13-01076-s001.zip › jpm-2454680-Supplementary.pdf]

Table S1. Univariate log-rank analysis of overall survival.

Variable	N. of patient with data	Median survival, month	HR (95%CI)	p value
**Age at diagnosis, y**			3.3 (2.2–5.0)	<0.001
>60	133	69		
≤60	155	NR		
**Sex**			2.5 (1.7–3.7)	<0.001
Male	169	105		
Female	119	148		
**Stage**			NA	0.029
I	9	NR		
II	19	NR		
III	20	105		
IV	17	129		
**Lymphoma type**			NA	<0.001
B-cell lymphoma	185	113		
Classical Hodgkin lymphoma lymphoma	79	NR		
T-cell lymphoma	19	46		
Lymphoma, NOS	5	42		
**LDH at diagnosis**			1.3 (0.6–2.9)	0.12
Elevated	48	166		
Normal	65	NR		

HR : Hazard ratio ; NR : not reached ; NA : not applicable.

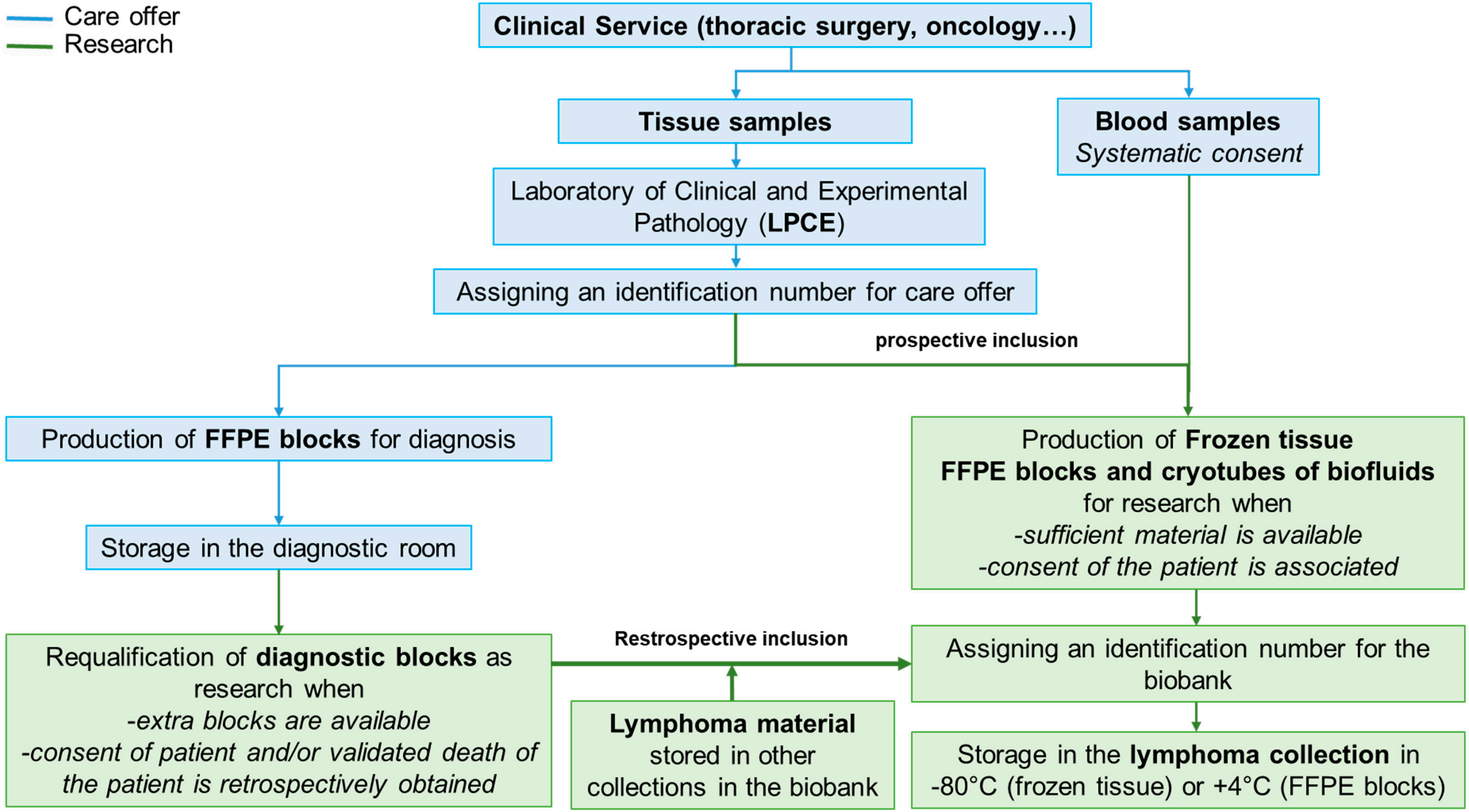

Supplemental Figure S1. Flow chart of the cohort of cases included in the lymphoma collection of the biobank
